# Identifying delirium in older adults presenting to a primary care out-of-hours (OOH) service: a retrospective cohort study

**DOI:** 10.1093/ageing/afag126

**Published:** 2026-05-11

**Authors:** Anna Elizabeth Seeley, Rachel Brettell, Ariel Wang, Rebecca Barnes, Sarah Tamsin Pendlebury, Gail Hayward

**Affiliations:** Nuffield Department of Primary Care and Health Sciences, Oxford University, Radcliffe Observatory Quarter, Woodstock Road, Oxford, Oxfordshire OX2 6GG, UK; Nuffield Department of Primary Care and Health Sciences, Oxford University, Radcliffe Observatory Quarter, Woodstock Road, Oxford, Oxfordshire OX2 6GG, UK; Nuffield Department of Primary Care and Health Sciences, Oxford University, Radcliffe Observatory Quarter, Woodstock Road, Oxford, Oxfordshire OX2 6GG, UK; Nuffield Department of Primary Care and Health Sciences, Oxford University, Radcliffe Observatory Quarter, Woodstock Road, Oxford, Oxfordshire OX2 6GG, UK; Wolfson Centre for Prevention of Stroke and Dementia, Nuffield Department of Clinical Neurosciences, University of Oxford, Wolfson Building Headington, Oxford, Oxfordshire OX3 9DU, UK; NIHR Oxford Biomedical Research Centre, Oxford University Hospitals NHS Foundation Trust, Headley Way Headington, Oxford, Oxfordshire OX3 9DU, UK; Nuffield Department of Primary Care and Health Sciences, Oxford University, Radcliffe Observatory Quarter, Woodstock Road, Oxford, Oxfordshire OX2 6GG, UK

**Keywords:** delirium, primary care, out of hours service, older adults

## Abstract

**Introduction:**

Out-of-hours (OOH) services provide urgent primary care outside normal General Practice (GP) hours, serving patients who cannot wait for routine service. Delirium is commonly associated with acute illness, causes distress and leads to poor outcomes. However, little is known about delirium presentations in OOH services. We aimed to investigate using records from an OOH service in South-West England.

**Methods:**

The database contained 33 345 contacts with patients ≥65 years attending the OOH service. We screened consultations during April and July 2019, and January 2020 using an automated search then clinical review by two independent GPs. We validated our search strategy by reviewing a random sample of 100 ‘search-negative’ consultations initially and assessed inter-rater reliability. Patient characteristics were compared using Chi-squared tests.

**Results:**

Of 4288 consultations with patients ≥65 years in the study periods, 402 (9.4%) involved possible or probable delirium. A further 74 (1.7%) had end-of-life delirium and were excluded from further analysis. Patients with delirium were older (mean age 84.4 years vs. 80.1 years), and more often had dementia (46.6% vs. 10.4%, *P* < .001). 67.9% of delirious patients required home visits, compared to 22.2% without delirium (*P* < .001). Patients with delirium were admitted to hospital twice as often as those without (20.6% vs. 8.5%, *P* < .001).

**Conclusions:**

Delirium is a common OOH presentation, representing ~10% of consultations with patients ≥65 years. These patients often have cognitive impairment, require home visits and are more likely to be admitted to hospital. These findings are important for planning urgent care services tailored to the needs of older people.

Key Points

- Symptoms of delirium commonly present to primary care out of hours services.
- The majority of those with symptoms of delirium require a home visit assessment.
- Those with possible or probable delirium are twice as likely to be admitted to hospital.

## Introduction

Delirium is a common severe acute neuropsychiatric condition affecting around one in four hospitalised older adults [1, 2] and is associated with multiple adverse outcomes including longer length of stay, mortality and risk of institutionalisation [3, 4]. One previous survey of older adults seen in a primary care ambulatory unit demonstrated that delirium was the most important risk factor for onwards referral to hospital [5]. Delirium is also an important marker of brain health: unmasking previously undetected cognitive impairment, as well as contributing to further cognitive decline, particularly for individuals with recurrent or prolonged episodes [6–9]. Of note, the 2019 Scottish Intercollegiate Guideline Network (SIGN) guidelines recommend all those who experience delirium have a follow-up assessment in primary care [10].

Historically, delirium has been under-recognised and underreported [11–13]. Thirty to sixty percent of delirium in secondary care is missed, despite numerous national guidelines [14] and policy initiatives [15] encouraging recognition, although recent evidence suggests that high rates of screening are achievable [4, 16]. Prevalence rates in otherwise well community-dwelling older adults are reportedly very low, around 0.5% [17]. However, this does not account for older adults presenting to primary care due to acute illness. Estimating this epidemiology is difficult. Delirium may not be routinely coded by primary care clinicians [18], nor are there any easy ways to identify older adults presenting with acute illness versus chronic problems. Prospective ascertainment is impractical given the relatively small and mixed patient population attending at individual surgeries.

Urgent care services, including GP Out of Hours (OOH) see patients at higher acuity than routine practice, or those who perceive their problems cannot wait until the next working day [19]. Delirium causes high patient distress and caregiver burden [20], and together with underlying acute illness, is often a motivating factor in health-seeking behaviour [21]. It is likely that a significant proportion of older adults presenting to OOH will have symptoms of delirium, yet this has never been estimated before, to our knowledge. It is also unknown how the presence of delirium affects clinical decision-making and outcome of the contact. In this study, we used data from an OOH service in South West England to retrospectively ascertain the occurrence of delirium in patients over 65 years then describe characteristics, diagnoses and outcomes of these OOH contacts.

## Methods

### Study population

This was a retrospective cohort review of electronic health records (EHR) for patients contacting a single OOH service provider between April 2019 and March 2020, serving a population of ~1 million people. We limited this study to contacts with patients aged ≥65 years. Ethics for original data collection was obtained as part of the Out of Hours Prescribing: Enhancing Communication (OPEN project), a mixed-methods study designed to explore diagnosis and treatment of infections in urgent care settings (Wales Reference Ethics Committee reference 18/WA/0413). Additional ethics for secondary data analysis was approved through Oxford University Central University Research Ethics Committee (reference R79465/RE001).

### Data

Data were extracted from the OOH EHR system, including coded data and free-text entries. Data were pseudonymised on entry to the database, but through use of unique identifier numbers it remained possible to calculate frequency of re-presentation to OOH services. Demographic data included age, sex and index of multiple deprivation (IMD) decile of patient. Service data consisted of origin and timing of call, contact type (either telephone call, or telephone call followed by in-person consultation at clinic base or at home) and contact outcome. Clinical data included clinical codes applied at the end of the contact, and free text data entered by the clinician(s) during the consultation. A clinician is unable to close a case without coding a diagnosis or outcome so these are anticipated to be complete. Free text data includes the history of the problem, any examination of the patient, any tests performed and the overall diagnosis and management of the patient. OOH clinicians in this service were able to access a patient’s primary care record, and key information, such as relevant GP-recorded past medical and medication history were commonly transferred into free text notes. A given patient could have multiple consultations over the study period and all consultations that took place in this period were included.

### Delirium search

No cases had a diagnostic code of ‘Delirium’ as this was not available within this OOH service’s code dictionary. Given the large number of cases, it was not feasible to review free text records by hand for every OOH contact to assess for delirium. We therefore developed an algorithm to identify possible cases of delirium by screening the free-text of OOH records.

One researcher (AES) who is a practicing GP and has prior clinical and research expertise in delirium, screened the first 200 records to identify word descriptors of delirium symptoms. These were mapped against each domain of the Diagnostic and Statistical manual of Mental Disorders (DSM)—5 diagnosis of delirium [22] (Supplemental Data File, Appendix S1). Word descriptors were combined to create a search strategy for all free text entries in each patient’s record. A minimum of one matched word in the search strategy was used to identify records for full case review.

### Cognitive impairment search

Given that delirium is both more common and more difficult to diagnose in people with underlying cognitive impairment [2], and that the impact is relatively more adverse in those without comorbid dementia [23], it was important to determine prevalence of cognitive impairment. We designed a search to detect these cases (Supplemental Data File, Appendix S2).

### Sample

**Figure 1:**
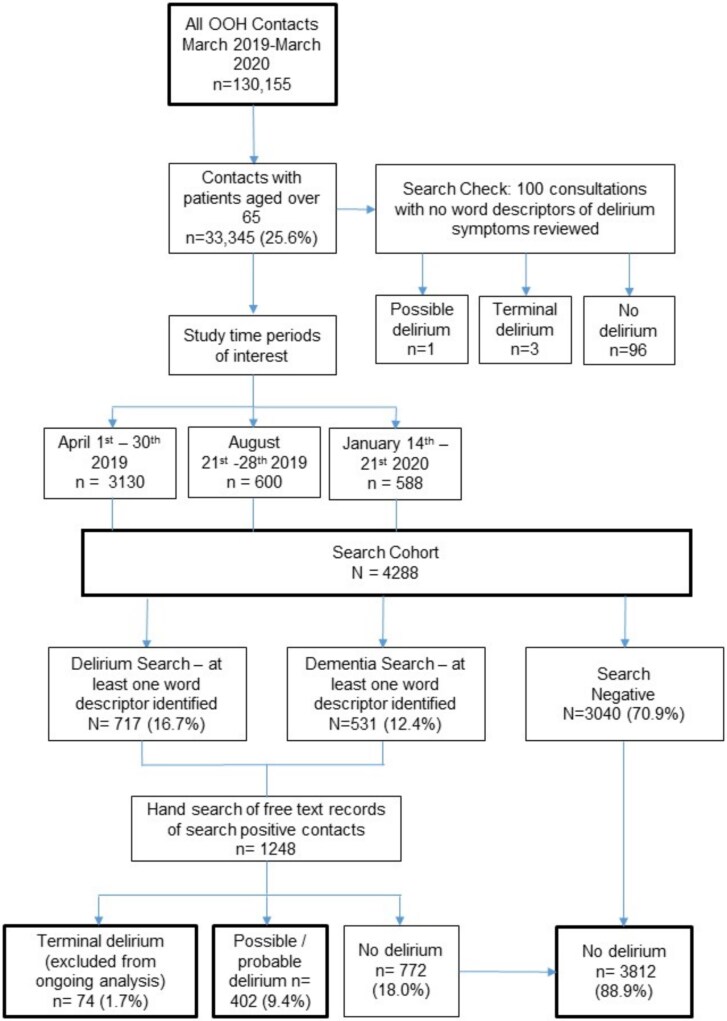
Study flow chart showing identification of delirium contacts presenting to the OOH service.

Given the size of the cohort, screening all consultations was not feasible owing to the time taken to complete the retrospective delirium assessments. Instead, we included all consultations with patients aged ≥65 years in April 2019, as we had 12 months of follow-up data to examine recurrent presentations. We also used a random date generator to identify a week in July (summer month) and January (winter month) given potential seasonal variation in delirium incidence [24, 25]. These three groups made up the study cohort (Figure 1). There were only two confirmed cases of COVID-19 in the UK by the end of January 2020, and so our results largely exclude the effect of the virus.

The ‘review cohort’ comprised all contacts delirium or dementia search positive within these three time periods. For all contacts within the review cohort, two GPs (AES and RB) hand reviewed the full consultation notes. Each case was scored using the DSM-5 diagnostic domains for delirium, and given an overall assessment of ‘Probable,’ ‘Possible,’ ‘Terminal’ or ‘No Delirium’ (Table 1), similar to methodologies used in a previously validated study of retrospective hospital records [26]. Consultation notes were also assessed by the same two clinicians for the presence of dementia or cognitive impairment.

**Table 1:** Results of clinician case review of contacts which were search positive within the study period.

Contacts identified via the delirium or dementia search	April 19 1st–30th (*n* = 936)	July 19 18th–24th (*n* = 166)	January 19 14th–20th (*n* = 146)	Total (*n* = 1248)
No delirium; *n* (%) No evidence of delirium on hand screening	573 (61.2%)	105 (63.3%)	94 (64.4%)	772 (61.9%)
Probable delirium; *n* (%) One of: Clinician makes a clear diagnosis of delirium Cases fulfils all five domains of DSM-V Very clear delirium (i.e. fulfils all other domains) in patients with dementia	76 (8.1%)	12 (7.2%)	18 (12.3%)	106 (8.5%)
Possible delirium, *n* (%) Fulfils some but not all of the DSM-V criteria Most patients with dementia in this category	232 (24.8%)	41 (24.7%)	23 (15.8%)	296 (23.7%)
Terminal delirium, *n* (%) Delirium symptoms but clearly linked to EoL presentation Identified through clear language about dying and/or prescription of anticipatory or palliative medication.	55 (5.9%)	8 (4.8%)	11 (7.5%)	74 (5.9%)

### Validation of search strategy

Initially, both clinicians individually reviewed the same 50 cases and compared results. Concordance was 80%. All discrepancies were discussed and agreement reached before continuing with the review process. All further cases where clinicians were unsure of a domain score or overall delirium assessment were discussed and agreement reached. Additionally, each researcher highlighted cases of uncertainty for discussion with STP, a senior academic geriatrician, with expertise in delirium.

We validated our search strategy by reviewing a random sample of 100 consultations classified as ‘search-negative.’ This search negative cohort included four ‘false negative’ delirium cases (three end of life delirium, one possible delirium). As a result of this, new terms ‘sleepy’ and ‘agitated’ were added to the search strategy to increase search sensitivity. All searches were re-run using the adapted search strategy.

### Software and statistical analysis

Data cleaning and data analysis were performed using R (version 4.5.2).

Descriptive statistics were used to describe the number and characteristics of older adults presenting to out of hours with no, possible and probable delirium and patient characteristics were compared using Chi-squared tests. A backwards selection logistic regression model was used to explore the factors associated with delirium. As missing data were very low (only for 3/3812 of those without delirium missing sex characteristics), a complete case analysis was used.

## Results

### Search results

In total, 130 155 contacts were made with the OOH GP service in the year between 1 April 2019 and 31 March 2020, with 33 345 contacts (25.6%) with patients aged ≥65 years. Figure 1 displays the study flow chart of cases searched and then reviewed. There were a total of 4288 contacts with patients ≥65 years within the study period, of which 1248 (29.1%) were positive for at least one word descriptor of delirium in our search, and thus hand-reviewed. Table 1 shows the breakdown of delirium subtypes identified for each time period. Frequency of delirium was broadly similar in each time period. Excluding those with terminal delirium from the rest of our analysis left 4214 contacts of which 402 (9.5%) had possible or probable delirium and 3812 (90.5%) without delirium. Possible delirium (296/4214, 7.0%) was over twice as common as probable delirium (106/4214, 2.5%).

### Individual patient characteristics

Within the study period 2490 individual patients were seen, of which 290 contacted the service at least once with possible or probable delirium. Characteristics of these patients are detailed in Table 2. Patients who presented with delirium were slightly older than those without (mean age 84.4 years vs. 80.1 years, *P* < .001), and there was a higher proportion of women (64.8% vs. 57.6%, *P* = .02).

**Table 2:** Individual patient characteristics of those presenting to the OOH service across the whole study period, in the search cohort without versus with delirium.

	All patients (*n* = 20 883)	Patients in search cohort with no delirium (*n* = 2151)	Patients in search cohort with delirium (*n* = 290)
**Patient characteristics**			
***Sex***			
Female; *n* (%)	12 156 (58.2%)	1239 (57.6%)	188 (64.8%)
Male; *n* (%)	8661 (41.6%)	910 (42.3%)	102 (35.2%)
Missing; *n* (%)	66 (0.2%)	2 (0.1%)	0 (0%)
***Age***			
Mean age in years (SD)	80.2 (8.80)	80.1 (8.7)	84.4 (8.0)
Age range in years	65–108	65–104	67–99
***Deprivation***			
Mean IMD (range)	6 (0–10)	6 (0–10)	6 (1–10)
***Dementia***			
	NA^a^	224 (10.4%)	135 (46.6%)

All patients: all patients aged ≥65 years contacting the service between 1 April 2019–31 March 2020.

Patients in search cohort: all patients aged ≥65 years contacting the service in April 2019, 18–24 July 2019 and 14–20 January 2020.

Patients in search cohort with delirium: all patients in the search cohort with a final diagnosis of possible or probable delirium.

^a^Data on dementia prevalence not available for all patients as this was determined through hand review.

### Contact characteristics

Contact characteristics during the study period are detailed in Table 3. Patients with possible or probable delirium were from a nursing or care home in 29.4% (118/402) of cases, double the frequency of those with no delirium (14.1%, 536/3812), but this was not statistically significant once accounting for age, sex and cognition [Supplemental Data File, Appendix S3, adjusted odds ratio (aOR) 1.01, 95% confidence intervals (CI) 0.99–1.04]. There appeared to be little difference in the time of day, or day of the week that patients with possible or probable delirium presented, compared to those without. A higher proportion of the delirium cohort required a home visit than those without (273/402, 67.9% vs. 848/3812, 22.2%, aOR 1.16, 95% CI 1.14–1.18).

**Table 3:** Characteristics of OOH service contacts across the whole study period, in the search cohort without versus with delirium.

	All contacts with patients aged ≥65 years from the OOH service (*n* = 33 345)	All contacts with patients aged ≥65 years in the search cohort^a^ (*n* = 4214)	
		Contacts with no delirium (*n* = 3812)	Contacts with delirium (*n* = 402)
***Call origin***			
111 referrals	253 (0.8%)	35 (0.9%)	2 (0.5%)
Ambulance	3857 (11.6%)	411 (10.8%)	44 (10.9%)
Care home	5208 (15.6%)	536 (14.1%)	118 (29.0%)
Health care professional	4227 (12.7%)	479 (12.6%)	36 (9.0%)
Missing	19 161 (57.5%)	2287 (60.0%)	196 (48.8%)
Other	61 (0.2%)	8 (0.2%)	0 (0%)
Patient line	570 (1.7%)	56 (1.5%)	6 (1.5%)
***Day of week***			
Weekday	8469 (25.4%)	985 (25.8%)	80 (19.9%)
Weekend	23 300 (69.19%)	2370 (62.2%)	276 (68.7%)
Bank holiday	1573 (4.7%)	457 (12.0%)	46 (11.4%)
***Time of day***			
8:00 a.m.–17:59 p.m.	16 583 (49.7%)	1941 (50.9%)	229 (57.0%)
18:00 p.m.–21:59 p.m.	8514 (25.5%)	946 (24.8%)	108 (26.9%)
22:00 p.m.–07:59 a.m.	8248 (24.7%)	925 (24.3%)	65 (16.1%)
***Type of contact***			
Clinician advice	16 176 (48.5%)	1828 (48.0%)	78 (19.4%)
F2F appointment	5748 (17.2%)	820 (21.5%)	33 (8.2%)
Follow up	1140 (3.4%)	113 (3.0%)	16 (4.0%)
Home visit	8056 (24.2%)	848 (22.3%)	273 (67.9%)
Other	144 (0.4%)	6 (0.2%)	0 (0%)
Repeat medication	1458 (4.4%)	197 (5.2%)	2 (0.5%)
No record/missing	623 (1.9%)	0 (0%)	0 (0%)
***Diagnostic codes***			
Non-specific	8881 (26.6%)	1068 (28.0%)	105 (26.1%)
Confusion	294 (0.9%)	2 (0.05%)	28 (6.9%)
All infection	7286 (21.9%)	741 (19.4%)	192 (47.8%)
Urinary tract infection	2442 (7.3%)	228 (6.1%)	93 (23.1%)
Lower respiratory tract infection	2378 (7.1%)	205 (5.4%)	79 (19.7%)
***Outcome of contact***			
Self-care	19 846 (59.5%)	2397 (62.9%)	259 (64.3%)
Referral to own GP	6347 (19.0%)	638 (16.7%)	55 (13.7%)
Referral to hospital	3095 (9.3%)	325 (8.5%)	83 (20.6%)
Patient deceased	1609 (4.8%)	195 (5.1%)	0 (0%)
Community referral	537 (1.6%)	81 (2.1%)	5 (1.2%)
Outpatient referral	128 (0.4%)	21 (0.6%)	0 (0%)
Did not attend/failed contact	353 (1.1%)	0 (0%)	0 (0%)
No record/missing	1430 (4.3%)	16 (0.4%)	0 (0%)
***Antibiotic prescription***	2561 (7.7%)	380 (10.0%)	75 (18.7%)

All contacts in the search cohort: all contacts with patients aged ≥65 years in April 2019, 18–24 July 2019 and 14–20 January 2020.

Contacts in search contact with no delirium: all contacts from the search cohort where no diagnosis of possible or probable delirium was made.

Contact in search cohort with delirium: all contacts from the search cohort with a final diagnosis of possible or probable delirium.

^a^Excluding those with terminal delirium.

### Coded diagnoses

Non-specific diagnostic codes, which included ‘Other reasons for encounter’ and ‘Advice about treatment given’ were commonly used, comprising 26.2% (105/402) of patients with delirium. Confusion was rarely coded, used in only 6.9% (28/402) of those with delirium. Infection codes were notably higher in the delirium cohort, with the frequency of urinary tract infection (UTI) codes particularly high compared to those without delirium (93/402, 23.1% vs. 228/3812, 6.1%, *P* < .001).

### Contact outcomes

Patients with delirium were referred to hospital from OOH twice as often as those without (83/402, 20.6% vs. 325/3812, 8.5%, *P* < .001). The majority of delirium consultations (63.2%), as with non-delirium consultations (63.0%) were discharged from the OOH service with ‘Self-Care,’ and a similar number flagged for GP follow-up (55/402, 13.7% for in the delirium cohort versus 638/3812, 16.7% in the non-delirium cohort, *P* = .33). Rates of community referral were low (1.3% in delirium cohort, 2.1% in those without delirium), and this was usually to district nursing team. Only one patient with delirium and two without were referred to the ‘Rapid Response Team,’ an advanced practitioner led service which could provide additional monitoring and treatment at home. Nineteen percent (75/402) were prescribed antibiotics in the delirium cohort, compared with 10% (380/3812) in those without (*P* = .001).

### Cognitive impairment, care home residence and delirium

**Figure 2:**
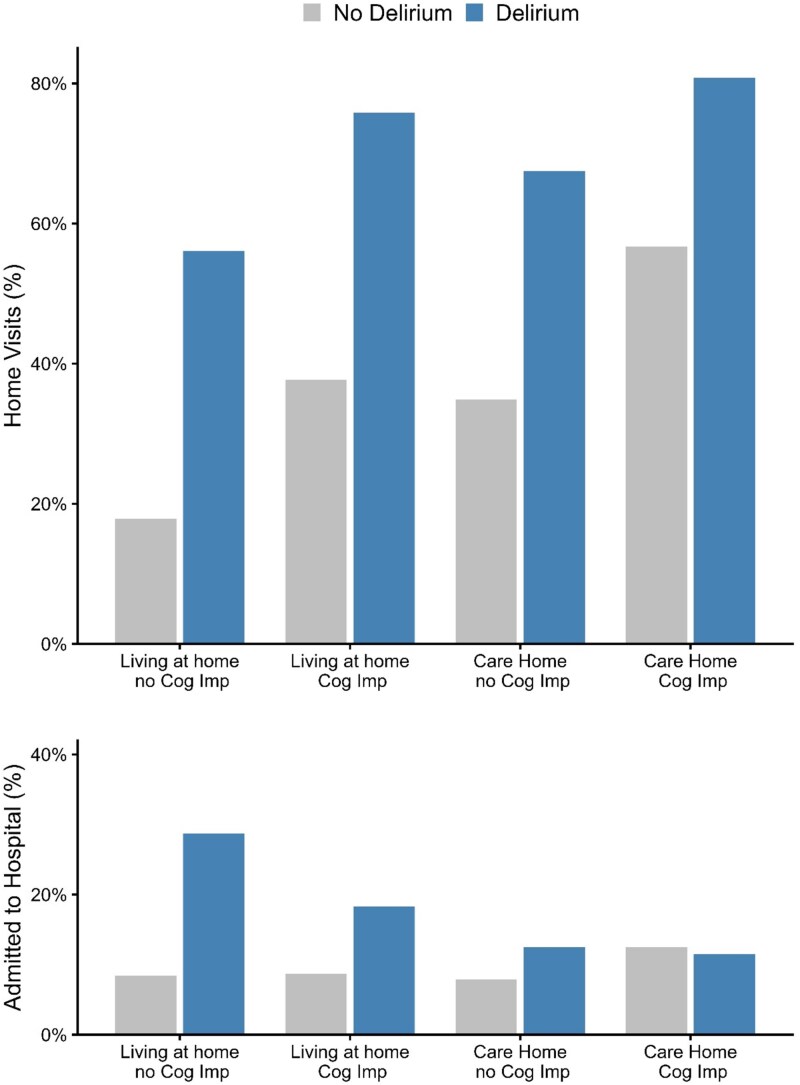
Home visits and hospital admission subgrouped by residency and cognitive status. Cognitive impairment (Cog Imp) was defined by any documented history of known cognitive impairment or dementia.

Supplemental Data File, Appendix S4 compares characteristics of those with probable or possible delirium, versus no delirium, subgrouped by the presence/absence of cognitive impairment including known dementia, (*n* = 570) and care home residency (*n* = 654). Delirium was seven times more prevalent in patients with a prior diagnosis of cognitive impairment (198/570, 34.7% vs. 204/3644, 5.6%, *P* < .001). Over 50% of patients with delirium symptoms required a home visit, regardless of cognitive impairment or care home residency, but the highest proportion was for care home residents with cognitive impairment (63/78, 80.8%) (Figure 2). Patients living at home were more likely to be admitted when delirium symptoms were present, and for those without cognitive impairment the admission rate from home was over three times higher with delirium than without (47/164, 28.7% versus 255/3024, 8.4%). In contrast, there was a much smaller difference in admission rate for patients living in a care home with and without delirium (Figure 2).

### Recurrent attendances

We were able to examine recurrent attendances over 12 months for patients who contacted OOH in April 2019. Those without delirium re-presented slightly more often than those with delirium over the following year [2.02 (SD 7.28) vs. 1.5 (SD 3.93), *P* = .047], but we were not able to account for survivorship or place of care in this period. We examined the proportion of patients who returned within 7 days for the entire search cohort. There was no difference between those with possible/probable delirium (49/402, 12.2%) and those without delirium (502/3812, 13.2%), (*P* = .68). Results did not change when we excluded those who were admitted to hospital on the first presentation (Supplemental Data File, Appendix S5).

## Discussion

### Summary of results

In this descriptive analysis of a large cohort of OOH consultations, symptoms of delirium were common, affecting around 10% of all consultations with older adults. Despite this ‘Delirium’ could not be coded within this service’s EHR, and alternative codes, such as ‘Acute Confusion’ were rarely used. The requirement for home visits was significantly higher for those with possible or probable delirium, after accounting for age and residency, representing substantial service demand. Notably, hospitalisation with delirium was highest for those living at home, particularly for those without pre-existing cognitive impairment. This may be related to delirium as a marker of illness severity, but also the capacity of relatives to care for those with delirium outside an institutionalised setting.

### Comparison to other literature

In another OOH service evaluation in a neighbouring county in England, the hospitalisation rate was 8.1% [27]. This is similar to our findings across the entire cohort, suggesting our patient population may be broadly comparable to other OOH services. A previous study of older adults contacting an OOH service in Ireland found 25% required home visits [28], almost the same as 24.2% of the entire cohort in this study, and found that older adults generally only contacted OOH services when seriously unwell. Our estimate of delirium prevalence supports this finding, sitting between primary and secondary care estimates. In the only community-based study of delirium prevalence 0.5% [17] were identified, whereas in secondary care rates are estimated at 23%–33% [1, 2].

Previous research from the Netherlands demonstrated that higher rates of frailty, female gender and nursing home residence were all associated with high rate of OOH use [29]. The relatively high number of delirium-related consultations and care home contacts in our study points to a substantial burden of frailty and complexity managed by OOH clinicians. However, we found scant evidence that clinicians were utilising any services which could provide additional support or monitoring in the community during the acute illness episode. This chimes with other evidence from urgent and emergency care that services designed to mitigate hospital admission for patients living with frailty or complex multimorbidity are not accessible ‘out of hours’ [30].

### Implications for clinical practice

Our findings may inform future service planning and delivery. Delirium symptoms were identified in 10% of older adult consultations, and these contacts typically required a home visit for further assessment. As out-of-hours home visits are resource intensive, services must be appropriately staffed and equipped to meet this demand. Patients with delirium living in care homes were admitted less frequently than those living at home, which may reflect greater capacity to manage delirium in care homes, differences in illness severity or the presence of advanced treatment decisions. Since these data were collected, acute frailty wards and hospital-at-home services [31] have expanded, alongside renewed policy emphasis on delivering care for older people outside hospital settings [32]. Patients with delirium—particularly those with pre-existing cognitive impairment, in whom delirium may be triggered by minor precipitants and where hospital admission may worsen symptoms [33]—represent a potential target group for such services. Further research is needed to determine the level and type of community support required to safely manage delirium in different patient groups.

The majority of patients in our study with possible or probable delirium were managed by the OOH clinicians in their usual place of residence. The downstream effects of community-based delirium are uncertain, as cognitive outcomes in delirious patients have predominantly been studied in in-patients. SIGN recommend patients who experience in-patient delirium are followed up in primary care [10]. While this may not be feasible or necessary for all those who do not require admission, those without pre-existing cognitive impairment, and who are relatively younger and more robust, potentially have the greatest amount to lose [2, 9, 34], and may constitute a group where targeted follow-up is warranted.

### Strengths and limitations

To our knowledge, this is the first description of delirium epidemiology within GP OOH services. We had access to consultations from a well-established service, covering 1 million patients. Crucially, we were able to integrate the clinician’s free text notes. Our finding that no consultations were coded as delirium demonstrates that this study could not be performed with coded EHRs alone.

This study has several limitations. Our study was based on a single OOH service and data were collected prior to COVID-19 pandemic. Given there is regional variation in how OOH services are organised and there has been a subsequent national roll-out of virtual wards and neighbourhood teams, it is difficult to know how generalisable our findings are. Retrospective ascertainment of delirium is only an estimate of the true occurrence. We based our methods on a previously validated study, which demonstrated a sensitivity of 0.58 and specificity of 0.93 for probable delirium, and 0.89 and 0.75 for possible delirium [24]. However, this study was of in-patient medical notes with clinical observations available over multiple time-points. In contrast, we only had a snapshot, and our assessment of that symptoms were acute and fluctuating was reliant on a documented collateral history. It may therefore be the case that some patients had subsyndromal delirium where some, but not all the DSM-5 criteria were met, particularly in the possible delirium group. Conversely, some subsyndromal delirium may not have presented to OOH owing to mild symptoms and less severe underlying illness. From a clinical perspective, the distinction between delirium and subsyndromal delirium may not be so important since previous studies of subsyndromal delirium, or related entities including ‘uncertain’ delirium or transient cognitive impairment, have shown that outcomes including excess mortality and dementia are similar [35–37].

Our estimations were based on reviewing around 5% of the annual total contacts with patients aged over 65. It was impractical to review all cases due to the time this would have taken. We validated our search techniques in several ways: through sampling at three different time points, reviewing ‘search-negative’ cases and reviewing all those with cognitive impairment, given this constitutes a group at high risk. However, we will have inevitably missed some cases—so potentially underestimating our findings. Concordance between the two clinical assessors was good, but not perfect. However, delirium diagnosis is challenging, and diagnostic uncertainty is common even when prospective screening is undertaken [4]. We flagged all difficult cases for discussion, including with a senior geriatrician.

## Conclusions

Delirium is a common OOH presentation, representing ~10% of consultations with patients aged over 65 years. These patients often have cognitive impairment, require home visits and are more likely to be admitted to hospital. These findings are important for planning urgent care services tailored to the needs of older people.

## Supplementary Material

afag126_Supplementary_materials

## Data Availability

Data were obtained through an NHS Out of Hours Service as part of the OPEN Project. Ethical approval was provided through Wales Reference Ethics Committee (reference 18/WA/0413). Additional ethics for secondary data analysis was approved through Central University Research Ethics Committee (reference R79465/RE001). Data cannot be shared publicly by the authors due to restrictions on use and storage, as specified within the ethical approval, and general conditions on use of NHS data. The authors will provide, on request, further summary level data, subject to approvals and conditions of use.
