# Supplementary material for: Identifying delirium in older adults presenting to a primary care out-of-hours (OOH) service: a retrospective cohort study
Source: Age Ageing. 2026 May 11;55(5):afag126. doi: 10.1093/ageing/afag126 (PMC13162239; doi:10.1093/ageing/afag126)
Supplement: afag126_Supplementary_materials [file afag126_supplementary_materials.docx]

**Supplemental Data File for *Identifying delirium in older adults presenting to a primary care out-of-hours (OOH) service: a retrospective cohort study.***

[Appendix 1: Word Descriptors identified from clinical records which were used by clinicians to document symptoms of delirium, based on the DSM-5 diagnostic domains of delirium. 2](#_Toc223682991)

[Appendix 2: Search terms for dementia and cognitive impairment screen 4](#_Toc223682992)

[Appendix 3: Forest plot of presenting characteristics associated with possible or probable delirium. 5](#_Toc223682993)

[Appendix 4: Variations between contacts with delirium and without depending on the presence of co-morbid cognitive impairment (CI) and care home residency 6](#_Toc223682994)

[Appendix 5: Reattendances to the OOH service after initial contact within 7 days, by presence of delirium symptoms. 7](#_Toc223682995)

[References 8](#_Toc223682996)

# **Appendix 1: Word Descriptors identified from clinical records which were used by clinicians to document symptoms of delirium, based on the DSM-5 diagnostic domains of delirium.**

The word descriptors chosen adopted a broad definition and included symptoms identified in a recent systematic review of sub-syndromal delirium,^1^ i.e. which otherwise might not be identified using a strict definition of DSM-V criteria.

| **Domain** | **Definition** | **Search Terms** |
| --- | --- | --- |
| Domain 1 | A disturbance in attention - reduced ability to direct, focus, sustain and shift attention | distract* , inattent* |
|  | A disturbance in awareness (reduced orientation to the environment) | DLOC, drowsy, agitat*, aggress*, glazed, |
| Domain 2 | The disturbance develops over a short period of time (usually hours to days) | No search terms due to variability in written history, scored on case review |
|  | Represents a change from baseline attention and awareness | altered behaviour, change in behaviour, behavioural change  Also scored on case review given variability in describing new symptoms |
|  | Tends to fluctuate in severity during the course of the day | sundown |
| Domain 3 | An additional disturbance in cognition (e.g. memory deficit, disorientation, language, visuospatial ability or perception | conf*, strange, mudd*, deliri*, deleri*, hallucinate*, out of sorts    Additionally any records with a coded diagnosis of ‘Confusion’ were screened in at this step. |
| Domain 4 | Exclusion: better explained by a pre-existing, established or evolving neuro-cognitive disorder | dement*, alzh*, cog* impair*, memory problem, |
|  | Exclusion: occurs in the context of severely reduced level of arousal such as coma | (minority of OOH patients) - GCS 3 |
| Domain 5 | Evidence from the history, physical examination or laboratory findings that the disturbance is a direct physiological consequence of another medical condition, substance intoxication or withdrawal, or exposure to a toxin, or is due to multiple aetiologies | Coded diagnosis and/or clinician freetext diagnosis and judgement |

Each contact contained up to 10 fields of free text notes entered by the clinicians assessing the patient. Each contact only had one entry, multiple clinicians could document within this contact. However patients may have multiple contacts if they presented on multiple different occasions to the OOH Service.

The algorithm worked by searching for each Search Term across all free text fields. A contact was ‘Search positive’ if at least one Search Term was detected in at least one field. Negative search terms such as ‘No Confusion’ were not used to rule out contacts for review as there was too much variation in how negative findings were documented and it did not allow for fluctuation in the clinical presentation.

# **Appendix 2: Search terms for dementia and cognitive impairment screen**

| **Search terms for dementia** | **Search terms for cognitive impairment** |
| --- | --- |
| dement*  alzh*  lewy  lbd | cognitive impairment  impaired cognition  cog* impair*  memory problem |

# **Appendix 3: Forest plot of presenting characteristics associated with possible or probable delirium.**

Plotted is the adjusted Odds Ratio (OR) with 95% Confidence Intervals (CI). The dashed line is the line of null effect. For categorical variables the reference variable (ref) is represented with an OR of 1.0.


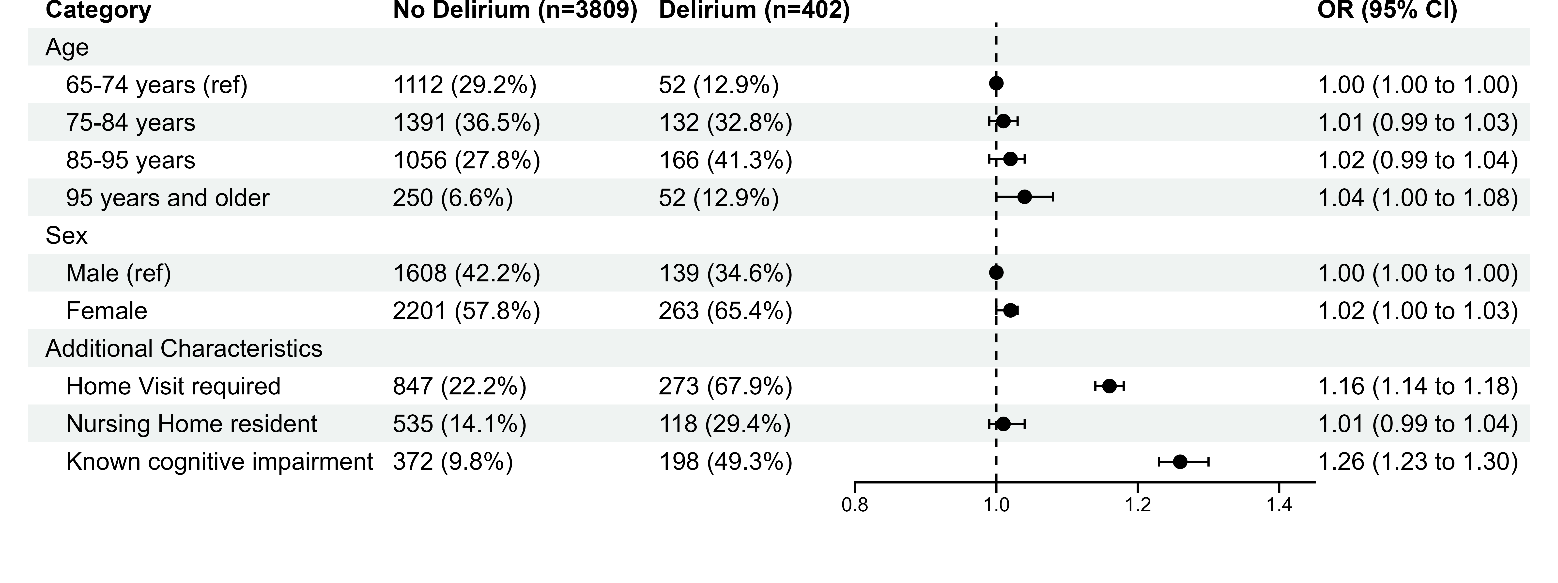


# **Appendix 4: Variations between contacts with delirium and without depending on the presence of co-morbid cognitive impairment (CI) and care home residency**

|  | **Living at home (n=3560)** | | | | **Care Home residents (n=654)** | | | |
| --- | --- | --- | --- | --- | --- | --- | --- | --- |
|  | **No CI, living at home (n=3188)** | | **CI, living at home (n=372)** | | **No CI, Care Home resident (n=456)** | | **CI, Care Home Resident (n=198)** | |
|  | *No Delirium (n=3024)* | *Delirium (n=164)* | *No Delirium (n=252)* | *Delirium (n=120)* | *No Delirium (n=416)* | *Delirium (n=40)* | *No Delirium (n=120)* | *Delirium (n=78)* |
| **Home Visit (%)** | 540 (17.9%) | 92 (56.1%) | 95 (37.7%) | 91 (75.8%) | 145 (34.9%) | 27 (67.5%) | 68 (56.7%) | 63 (80.8%) |
| **Admission to Hospital (%)** | 255 (8.4%) | 47 (28.7%) | 22 (8.7%) | 22 (18.3%) | 33 (7.9%) | 5 (12.5%) | 15 (12.5%) | 9 (11.5%) |
| **Coded Diagnosis UTI (%)** | 204 (6.7%) | 34 (20.7%) | 16 (6.3%) | 31 (25.8%) | 10 (2.4%) | 14 (35.0%) | 4 (3.3%) | 15 (19.2%) |

# **Appendix 5: Reattendances to the OOH service after initial contact within 7 days, by presence of delirium symptoms.**

Numbers shown are for the whole cohort and then removing those who were admitted to hospital.

|  | **Patients who did not present with possible or probable delirium** | **Patients who had a presentation with possible or probable delirium** |
| --- | --- | --- |
| *All contacts in the search cohort (n=4214)* | | |
| Number of patients | 3812 | 402 |
| Number who returned to OOH service within 7 days (%) | 502 (13.2%) | 49 (12.2%) |
| *Contacts where final outcome of the index contact was not admission to hospital (n= 3878)* | | |
| Number of patients | 3487 | 319 |
| Number who returned to OOH service within 7 days (%) | 482 (13.8%) | 43 (13.5%) |

# **References**

1. Bowman, E. M. L. *et al.* Assessment and report of individual symptoms in studies of delirium in postoperative populations: a systematic review. *Age Ageing* **53**, 77 (2024).
